# Study protocol for a randomised controlled trial to investigate the effectiveness of thoracic epidural and paravertebral blockade in reducing chronic post-thoracotomy pain: 2 (TOPIC 2)

**DOI:** 10.1186/s13063-023-07463-1

**Published:** 2023-11-23

**Authors:** Ben Shelley, Andreas Goebel, Stephen Grant, Louise Jackson, Hugh Jarrett, Marcus Jepson, Amy Kerr, Nandor Marczin, Rajnikant Mehta, Teresa Melody, Lee Middleton, Babu Naidu, Lajos Szentgyorgyi, Sarah Tearne, Ben Watkins, Matthew Wilson, Andrew Worrall, Joyce Yeung, Fang Gao Smith

**Affiliations:** 1 School of Medicine, University of Glasgow, Glasgow, UK; 2 Institute of Translational Medicine, University of Liverpool, Liverpool, UK; 3 Health Economics Unit, University of Birmingham, Birmingham, UK; 4 Birmingham Clinical Trial Unit, Birmingham, UK; 5 Population Health Sciences, University of Bristol, Bristol, UK; 6 University Hospitals Birmingham Thoracic Surgical Research Centre, Bristol, UK; 7 Department of Surgery and Cancer, Imperial College London, London, UK; 8 University Hospitals Birmingham, Birmingham, UK; 9 Institute of Inflammation and Ageing, University of Birmingham, Birmingham, UK; 10 Manchester University NHS Foundation Trust, Manchester, UK; 11 School of Health & Related Research, University of Sheffield, Sheffield, UK; 12 Warwick Medical School, University of Warwick, Coventry, UK

**Keywords:** Thoracotomy, Thoracic surgery, Paravertebral block, Thoracic epidural blockade, Anaesthesia, Chronic pain

## Abstract

**Background:**

Thoracotomy is considered one of the most painful surgical procedures and can cause debilitating chronic post-surgical pain lasting months or years postoperatively. Aggressive management of acute pain resulting from thoracotomy may reduce the likelihood of developing chronic pain. This trial compares the two most commonly used modes of acute analgesia provision at the time of thoracotomy (thoracic epidural blockade (TEB) and paravertebral blockade (PVB)) in terms of their clinical and cost-effectiveness in preventing chronic post-thoracotomy pain.

**Methods:**

TOPIC 2 is a multi-centre, open-label, parallel group, superiority, randomised controlled trial, with an internal pilot investigating the use of TEB and PVB in 1026 adult (≥ 18 years old) patients undergoing thoracotomy in up to 20 thoracic centres throughout the UK. Patients (*N* = 1026) will be randomised in a 1:1 ratio to receive either TEB or PVB. During the first year, the trial will include an integrated QuinteT (Qualitative Research Integrated into Trials) Recruitment Intervention (QRI) with the aim of optimising recruitment and informed consent. The primary outcome is the incidence of chronic post-surgical pain at 6 months post-randomisation defined as ‘worst chest pain over the last week’ equating to a visual analogue score greater than or equal to 40 mm indicating at least a moderate level of pain. Secondary outcomes include acute pain, complications of regional analgesia and surgery, health-related quality of life, mortality and a health economic analysis.

**Discussion:**

Both TEB and PVB have been demonstrated to be effective in the prevention of acute pain following thoracotomy and nationally practice is divided. Identification of which mode of analgesia is both clinically and cost-effective in preventing chronic post-thoracotomy pain could ameliorate the debilitating effects of chronic pain, improving health-related quality of life, facilitating return to work and caring responsibilities and resulting in a cost saving to the NHS.

**Trial registration:**

NCT03677856 [ClinicalTrials.gov] registered September 19, 2018. https://clinicaltrials.gov/ct2/show/NCT03677856. First patient recruited 8 January 2019.

## Administrative information

Note: the numbers in curly brackets in this protocol refer to SPIRIT checklist item numbers. The order of the items has been modified to group similar items (see http://www.equator-network.org/reporting-guidelines/spirit-2013-statement-defining-standard-protocol-items-for-clinical-trials/).

**Table:** 

Title {1}	A randomised controlled trial to investigate the effectiveness of thoracic epidural and paravertebral blockade in reducing chronic post- thoracotomy pain: 2 (TOPIC 2).
Trial registration {2a and 2b}.	NCT03677856 [ClinicalTrials.gov] registered September 19^th^ 2018. https://clinicaltrials.gov/ct2/show/NCT03677856 First patient recruited 8^th^ January 2019.
Protocol version {3}	5.0 (8^th^ October 2021)
Funding {4}	This trial is funded by the UK National Institute of Health Research Health Technology Assessment Programme (NIHR-HTA), funder’s reference: 16/111/111.
Author details {5a}	Ben Shelley—School of Medicine, University of Glasgow; A Goebel – Institute of Translational Medicine, University of Liverpool; S Grant—Lay member; L Jackson—Health Economics Unit, University of Birmingham H Jarrett—Birmingham Clinical Trial Unit; University of Birmingham M Jepson—Population Health Sciences – University of Bristol; A Kerr—University Hospitals Birmingham Thoracic Surgical Research Centre; N Marczin—Dept of surgery and Cancer – Imperial College London; R Mehta—Birmingham Clinical Trial Unit; University of Birmingham T Melody—University Hospitals Birmingham; Lee Middleton—Birmingham Clinical Trial Unit; University of Birmingham B Naidu—Institute of Inflammation and Ageing, University of Birmingham (TOPIC-2 Surgical Lead); L Szentgyorgyi— Manchester University NHS Foundation Trust; S Tearne—Birmingham Clinical Trial Unit; University of Birmingham B Watkins—Birmingham Clinical Trial Unit; University of Birmingham M Wilson – School of Health & Related Research, University of Sheffield; A Worrall—Lay member; J Yeung—Warwick Medical School, University of Warwick; F Gao Smith—Institute of Inflammation and Ageing University of Birmingham (TOPIC-2 Chief Investigator).
Name and contact information for the trial sponsor {5b}	The University of Birmingham is the trial sponsor. Contact: University of Birmingham, Edgbaston, Birmingham, B15 2TT; researchgovernance@bham.ac.uk.
Role of sponsor {5c}	Besides providing peer review advice at the time of funding, the funder and sponsor played no role in the design of the study, in collection, analysis, and interpretation of data or in writing this manuscript.

## Introduction

### Background and rationale {6a}

#### Background

Thoracotomy surgery (most commonly performed to treat lung cancer) is considered one of the most painful surgical procedures and can cause chronic post-surgical pain lasting months or years postoperatively. The presence of chronic post-thoracotomy pain (CPTP), defined as pain that recurs or persists at least 2 months following surgery [1], has been reported to occur with an incidence as high as 50% [2]. CPTP can be severe and debilitating to patients, leading to wide-ranging impacts on functional activity and quality of life, more frequent general practitioner visits, anxiety, depression, time off sick and unemployment [3–5].

Aggressive management of acute pain resulting from thoracotomy may reduce the likelihood of developing chronic pain [6]. Two main analgesic techniques are commonly used for perioperative pain control during thoracotomy, thoracic epidural blockade (TEB) and paravertebral blockade (PVB), which both seek to block afferent nociceptive transmission at a spinal cord level preventing ascending transmission. Some suggest that by unilaterally blocking afferent nerve transmission at the paravertebral space, PVB may more completely block nociceptive transmission than TEB [7, 8]. This total blockade of nerve signals could remove the stimulus for ‘central sensitisation’, which underpins the formation of chronic pain pathways [9]. PVB therefore could be uniquely effective in preventing long-term pain [10], and there is evidence from a recent trial of the two techniques in breast surgery to support this premise [11]. In our pilot feasibility trial, pain scores were lower on average with PVB compared with TEB but a much larger trial is required to confirm this finding reliably [12].

TEB and PVB have been widely examined in terms of acute outcomes and short-term benefits in patients undergoing thoracotomy. Systematic reviews and meta-analyses support the use of either technique, with evidence that PVB provides equivalent analgesia to TEB for acute pain [6, 12–16]. Whilst major complication rates appear similar between the two techniques, PVB is associated with less urinary retention, hypotension and nausea/vomiting [12–14, 17]. A Cochrane review of 14 studies (698 participants) comparing the two techniques was however forced to conclude that there was insufficient data on chronic pain to allow a comparison for this endpoint [13].

#### Trial rationale

“What can we do to stop patients developing chronic pain after surgery?” was identified as a top 10 research priority by the James Lind Alliance through the Anaesthesia and Perioperative Care Priority Setting Partnership, involving 25 professional and 20 patients/carer stakeholder organisations in 2015 [18]. Clinicians and researchers therefore have a moral and scientific duty to investigate treatments to prevent or reduce chronic post-surgery pain. For over a decade, both TEB and PVB have been widely used internationally [19–21] for the prevention of acute post-operative pain following thoracotomy, but their comparative effects on chronic pain are unknown. Identifying cost-effective ways of preventing CPTP could reduce patient suffering, loss of productivity, disruption to employment and use of health care resources.

### Objectives {7}

#### Primary objective

To test the hypothesis that in adult patients undergoing elective open thoracotomy, the use of paravertebral blockade for peri-operative pain relief reduces the presence of chronic pain at 6 months post randomisation by at least 10% compared with thoracic epidural blockade.

#### Secondary objectives

- To compare the effectiveness of PVB versus TEB in terms of quality of life, neuropathic pain symptoms, symptoms of anxiety/depression and patient satisfaction up to 12 months following surgery
- To compare the effectiveness of PVB versus TEB in terms of acute pain control up to 72 h following surgery, incidence of post-operative major and minor complications and length of post-operative hospital stay
- To analyse the costs and effectiveness of PVB compared with TEB

### Trial design {8}

TOPIC 2 is a multi-centre, open-label, parallel group, superiority randomised controlled trial, with an internal pilot investigating the use of TEB and PVB in 1026 adult (≥ 18 years old) thoracotomy patients. Patients will be randomised in a 1:1 ratio to receive either TEB (standard treatment) or PVB (interventional treatment). In addition, during the first year, the trial will include an integrated QuinteT (Qualitative Research Integrated into Trials) Recruitment Intervention (QRI) with the aim of optimising recruitment and informed consent [22].

## Methods: participants, interventions and outcomes

### Study setting {9}

Patients under the care of participating surgical and anaesthetic care teams in up to 20 thoracic centres throughout the UK.

### Eligibility criteria {10}

#### Inclusion criteria

- Aged ≥ 18 years
- Elective open thoracotomy
- Able to provide written informed consent
- Willingness to complete trial questionnaires out to 12 months post randomisation

#### Exclusion criteria

- Contraindication to TEB or PVB, e.g. known allergy to local anaesthetics; infection near the proposed puncture site; coagulation disorders; thoracic spine disorders
- Rib/chest wall resection or planned pleurectomy
- Previous thoracotomy on the same side
- Median sternotomy within 90 days

### Who will take informed consent? {26a}

It will be the responsibility of the investigator, or suitably qualified delegate, as identified on the Site Signature and Delegation Log, to receive written informed consent for each participant prior to performing any trial related procedure. All consent procedures will adhere to Good Clinical Practice (GCP) guidance.

For patients participating in the Qualitative Research Integrated into Trials study, an additional ‘Audio-recording discussions and interviews consent form’ will be completed alongside the main trial consent process.

### Additional consent provisions for collection and use of participant data and biological specimens {26b}

Not applicable, no biological specimens are being taken.

## Interventions

### Explanation for the choice of comparators {6b}

Both TEB and PVB for the provision of analgesia to patients undergoing thoracotomy are widely practised in UK thoracic anaesthesia such that both trial interventions may be considered standard care [19, 23]. Whilst these interventions have been deemed equivalent in terms of acute analgesia and complications supporting their widespread use [13]), this trial seeks to address the specific uncertainties regarding their efficacy in preventing CPTP and cost-effectiveness.

### Intervention description {11a}

Local anaesthetic will be delivered by continuous infusion through an epidural or paravertebral catheter for a minimum of 48 h postoperatively in both intervention arms.

#### Intervention group: PVB

Participating institutions ‘usual practice’ of PVB; three single-shot injections, at appropriate spinal levels supplying the skin over the incision site, will be given before the start of surgery. A PVB catheter will then subsequently be placed under direct vision during surgery. A loading dose is given before chest closure followed by continuous paravertebral infusion for post-operative use.

#### Standard group: TEB

Participating institutions ‘usual practice’ of TEB; epidural catheter is inserted at the spinal level supplying the skin at the incision site, followed by test dose and a loading dose before the start of surgery. An epidural infusion is set up for use during the operation and for postoperative use.

In this intentionally pragmatic trial, some variation in technical aspects of block insertion is anticipated, both between experienced thoracic anaesthetists and surgeons, and between centres, as clinicians will use their judgement on the best insertion techniques for each individual patient. These include the following: insertion using ultrasound or landmark techniques, use of bupivacaine, levobupivacaine or ropivacaine and addition of opiate. This represents real-world variation in clinical practice and will not contribute to bias since randomisation will ensure balance across groups by centre. These variations in practice will be captured in the case report form.

Trial treatment and interventions (TEB or PVB) will be performed by thoracic anaesthetists or surgeons (consultants or senior trainees) with experience in the techniques, who have reviewed trial training material and confirmed that they are able to perform the techniques.

### Criteria for discontinuing or modifying allocated interventions {11b}

Provision of adequate postoperative analgesia is the primary aim of patient care and must not be compromised by trial participation. Where it has not been possible to perform the randomised technique allocation (e.g. due to practical inability to place an epidural catheter, or disruption of pleural anatomy such that PVB catheter cannot be sited), it is permissible to perform an alternative regional analgesic technique, including where this constitutes crossover between study groups. All such protocol deviations (and the explanatory reasons) will be recorded and reported. Patients will nonetheless be retained within their allocated treatment group for analysis according to the principles of ‘intention-to-treat’.

### Strategies to improve adherence to interventions {11c}

Adherence to study technique allocations was high in the pilot trial [12]. Protocol deviations will be monitored throughout the trial by the trial management group and data safety and monitoring committee. Where deviation rates appear excessive, contact will be made with site investigators. Educational material in the form of videos and the option for on-site teaching will be offered to sites to improve the consistency of TEB and PVB performance.

### Relevant concomitant care permitted or prohibited during the trial {11d}

The trial seeks solely to randomise between two regional analgesic techniques. Clinicians are encouraged to use these trial interventions as part of a multi-modal analgesic technique and to provide adjunctive analgesia as deemed appropriate.

### Provisions for post-trial care {30}

The clinical interventions used in the trial are performed at a single point in time and cannot be amended in any way once performed. As such, there is no need to provide continuing post-trial care other than as standard local practice. In the event of complications related to the performance of the study interventions, these will be managed as per routine care at participating sites.

### Outcomes {12}

#### Primary outcome

Presence of CPTP at 6 months post-randomisation. Participants will be asked to indicate their ‘worst chest pain over the last week’ on a 100-mm visual analogue scale (VAS; 0–100). Presence of CPTP will be defined as a VAS score greater than or equal to 40 mm indicating at least a moderate level of pain.

#### Secondary outcomes

- Complications of regional analgesia (failure of blockade, hypotension (systolic blood pressure (< 90 mmHg), inadequate pain relief, low respiratory rate (< 10/min), drowsiness, nausea and vomiting, urinary retention, itching, high block, post-dural puncture headache, vascular puncture, pleural puncture) until discharge from hospital
- Occurrence and severity of surgical complications until discharge from hospital; occurrence as defined by the European Society of Thoracic Surgeons dataset [24] and severity as defined by the Thoracic Morbidity and Mortality (TMM) classification [25]
- Post-operative pulmonary complications (PPCs) until discharge from hospital (as defined by the standardised endpoints in perioperative medicine: pulmonary complications [26]
- Critical care admission (levels 2 and 3)
- Mortality (reported for all deaths due to all causes)
- Analgesic use
- Acute pain in the 3 days following surgery (patient reported via VAS; Brief Pain Inventory [27]; (Table 1))
- Pain at hospital discharge (via VAS, BPI and Short-form McGill Pain Questionnaire 2 (SF-MPQ-2 [28] (Table 1))
- Chronic pain (via VAS, BPI and SF-MPQ-2, completed by the participant at 3, 6 and 12 months post randomisation (Table 1))
- Resource use and cost data (resource use intraoperatively, during and following hospital admission, completed by the research team at each site and via telephone interviews with the patient following discharge, as appropriate)
- General health-related quality of life (by EQ-5D-5L [29], completed by the participant at hospital discharge and at 3, 6 and 12 months (Table 1))
- Mental health state (measured by Hospital Anxiety and Depression Scale (HADS [30]), completed by the participant at hospital discharge and at 3, 6 and 12 months)
- Serious adverse events

**Table 1:** Data collection tools and corresponding outcomes

Collection tool	Outcome	Possible responses
Visual analogue scale (VAS)	Chronic phase: • Worst chest pain over the last week score (primary outcome) • Average chest pain over the last week score Acute phase: • Worst chest pain over the last 24 h score • Average chest pain over the last 24 h score	All 0–100 (higher = worse score)
Brief Pain Inventory questionnaire (BPI) [27]	Interference score	0–10 (higher = worse score)
Short Form McGill questionnaire (SF-MPQ-2) [28]	• Continuous pain subscale score • Intermittent pain subscale score • Neuropathic pain subscale score • Affective pain subscale score • Overall score	All 0–10 (higher = worse score)
Generic health-related quality of life questionnaire (EQ-5D-5L) [29]	• Index score • Thermometer score	(− 0.59 = worst outcome, 1.0 = best outcome) (0–100, higher = better)
Hospital Anxiety and Depression Scale questionnaire (HADS) [30]	• Depression score • Anxiety score	Both 0–21 (lower = better)
Likert Scale to assess satisfaction	• Satisfaction with pain therapy after surgery • Satisfaction with care provided by hospital	Very dissatisfied/dissatisfied/satisfied/very satisfied

### Participant timeline {13}

The participant timeline is shown in Table 2.

**Table 2:** Participant timeline

			**Acute phase**				**Chronic phase**			
**Visit**	**Screening**	**Baseline**	**Day0**^**A**^	**Day 1**^**B**^	**Day 2**^**B**^	**Day 3**^**B**^	**Hospital discharge**	**Month 3**	**Month 6**	**Month 12**
**Enrolment**										
Eligibility check	x									
Valid informed consent		x								
Randomisation^C^			x							
**Baseline medical and demographic data**										
Medical history		x								
Baseline clinical assessments^D^		x								
Lung function tests (FEV_1_, FVC, DLCO)^E^		x								
**Clinical data**										
Trial intervention			x							
Complications of regional anaesthesia^F^			x	x	x	x	x			
Resource use (analgesics use etc.)			x	x	x	x	x	x	x	x
Mortality check			x	x	x	x	x	x	x	x
Post-operative surgical complications			x	x	x	x	x			
Post-operative pulmonary complications			x	x	x	x	x			
SAE check			x	x	x	x	x	x	x	x
**Patient reported data**										
Pain questionnaires (VAS, BPI)		x		x	x	x	x	x	x^G^	x
Pain questionnaire (SF-MPQ-2)		x					x	x	x	x
Quality of life questionnaires (EQ-5D-5L and HADS)		x					x	x	x	x
Patient Satisfaction (patient questionnaire)							x	x	x	x
Out of pocket costs incurred by participants								x	x	x
Societal cost (productivity loss etc.)								x	x	x

*FEV1* Forced expiratory volume in 1 s, *FVC* Forced vital capacity, *DLCO* Diffusing capacity for carbon monoxide, *SAE* Serious adverse event, *VAS* Visual analogue score, BPI Brief pain inventory, *SF-MPQ-2* Short form McGill pain questionnaire

^A^Day 0 is defined as the day of the intervention (and surgery)

^B^Day 1 is first full calendar day (from 12 midnight) post-surgery, day 2 is second full calendar day, day 3 is third full calendar day

^C^Randomisation performed on day of surgery or working day prior to surgery

^D^To include: ASA grade, height and weight, ECOG, shortness of breath category, smoking status, alcohol consumption. These should be assessed within 28 days of the intervention

^E^Lung function data can be taken from assessment within past 6 months prior to the intervention

^F^To include the following: failure of blockade, hypotension (systolic blood pressure (< 90 mmHg)), inadequate pain relief, low respiratory rate (< 10/min), drowsiness, nausea and vomiting, urinary retention, itching, high block, post-dural puncture headache, vascular puncture, pleural puncture

^G^Includes primary outcome—‘worst chest pain over the last week’ on a 100-mm visual analogue scale

### Sample size {14}

Assuming a 30% incidence of CPTP in the TEB group (similar to that seen in our previous TOPIC-pilot trial results [12] and systematic review [13], 392 patients in each group will give 90% power (two-sided, *p* = 0.05) to detect a 10% absolute reduction (i.e. down to 20%, a 33% relative reduction) in the PVB group. Assuming a 10% rate of death by 6-month follow-up (similar to that seen in the TOPIC pilot) and a further 15% loss to follow-up at 6 months, we will recruit a total of 1026 participants.

### Recruitment {15}

Patients will be recruited by reviewing the thoracic surgical lists of up to 20 large UK tertiary referral thoracic surgical centres with a track record of successful recruitment to clinical trials and an appropriate patient case mix.

## Assignment of interventions: allocation

### Sequence generation {16a}

Participants will be randomised in a 1:1 ratio to either TEB or PVB. A minimisation algorithm will be used within the online randomisation system to ensure balance in the treatment allocation over the following variables:

- Gender
- Age < 65 years or ≥ 65 years
- Centre
- Thoracotomy for lung cancer resection or for other indication

A ‘random element’ will be included in the minimisation algorithm, so that each participant has a probability (1:5), of being randomised to the opposite treatment that they would have otherwise received.

### Concealment mechanism {16b}

Randomisation will be provided by a secure online randomisation system at the Birmingham Clinical Trials Unit (BCTU) (available at https://www.trials.bham.ac.uk/TOPIC2).

### Implementation {16c}

After participant eligibility has been confirmed and informed consent has been received, the participant can be randomised into the trial. The patient should ideally be randomised on the day of surgery or the working day prior to surgery. Only when all eligibility criteria have been confirmed and all minimisation data items have been provided will randomisation take place and a trial number be allocated.

## Assignment of interventions: blinding

### Who will be blinded {17a}

Due to the nature of the intervention, attempts to completely blind clinicians would make processes prohibitively complex and expensive due to innate differences in the mode of action of the two analgesic techniques. There are treatment implications for the patients following their allocated procedure; therefore, the research staff and treating clinicians will be aware of the intervention received.

With regard to patients, the proposed primary outcome of pain rating is subjective in nature and the presence of detection bias is a theoretical possibility. However, there is no reason to suspect that recipients of the randomised interventions have strong preconceptions about the relative effectiveness of each analgesic technique. Furthermore, the primary outcome will be collected via questionnaires administered by post or telephone, at a time remote from the original operative procedure, which are therefore likely to be resilient to the effects of imperfect concealment. The trial participants will not be explicitly informed of the intervention allocation. In the pilot study, it was however acknowledged that it is difficult to maintain this blinding throughout their stay in hospital.

### Procedure for unblinding if needed {17b}

Not needed—study is not blinded.

## Data collection and management

### Plans for assessment and collection of outcomes {18a}

Case report forms will be completed for each individual subject according to Table 3.

**Table 3:** Case report forms and data collection

Form name	Contents
Baseline Medical Data Form	Height, weight, ECOG status, ASA grade, performance status, medical history, smoking status and history, lung function,
Patient Completed Booklet Hospital Baseline	Pain questionnaires: VAS, BPI and SF-MPQ-2. QoL – EQ-5D-5L, HADS,
Intervention Form	Analgesia administered in theatre and recovery, intraoperative monitoring, analgesic intervention performed, difficulties and complications with block, local anaesthetic administered
Operation Details Form	Operation type, operator, side, approach, muscle/nerve sparing, rib resection or fractures, chest drainage, histology
Acute Phase patient completed booklet (Day 1^A^, Day 2^A^, Day 3^A^)	Pain questionnaires: VAS and BPI
Acute Day 0^A^ Post Recovery Form	Management of LA block—top-ups, resiting and discontinuation. Analgesia administered. Return to theatre
Acute Phase Up To Day 3^A^ Form	Ward location. Management of LA block—top-ups, resiting and discontinuation. Analgesia administered. Complications and severity (Table 3), return to theatre
Acute Phase Day 4 to Discharge Form	Postoperative analgesia administered. Complications and severity (Table 3), return to theatre
Patient Completed Booklet Hospital Discharge	Patient satisfaction questionnaire. Pain questionnaires: VAS, BPI and SF-MPQ-2. QoL – EQ-5D-5L. HADS
Chronic Phase patient completed booklet (3, 6 and 12 months)	Patient satisfaction questionnaire. Pain questionnaires: VAS, BPI and SF-MPQ-2. QoL – EQ-5D-5L. HADS
Health Contacts Form (3, 6 and 12 months)	NHS Care visits: A&E visits, hospital admissions and investigations received Private healthcare costs. Medications and equipment. Occupation and activities

*ASA grade* American Society of Anaesthetists, *BPI* Brief pain inventory, *ECOG* Eastern Cooperative Oncology Group, *HADS* Hospital Anxiety and Depression Scale, *QoL* Quality of life, *SF-MPQ-2* Short form McGill pain questionnaire, *VAS* Visual analogue score

^A^Day 0 is defined as the day of the intervention (and surgery), Day 1 is first full calendar day (from 12 midnight) post-surgery, day 2 is second full calendar day, day 3 is third full calendar day

### Primary outcome data collection

Data collected from participants’ completed questionnaires forms the basis of the primary outcome. Questionnaires will be posted directly to the participant by the local site with a self-addressed envelope to enable the return of the questionnaires directly to the TOPIC 2 Trial Office. Participants will be asked to indicate their ‘worst chest pain over the last week’ on a visual analogue scale (VAS; 0–100). Presence of CPTP will be taken to be a score greater than or equal to 40 (mm) indicating at least a moderate level of pain.

### Plans to promote participant retention and complete follow-up {18b}

Throughout the recruitment and follow-up period, retention will be constantly assessed by the trial management group including patient representatives. A key strategy implemented to improve follow-up will be the provision of a thank you card and trial branded pen with the 6-month questionnaire to encourage completion of the questionnaire booklet which informs the primary outcome.

### Data management {19}

A secure database containing trial-related data will be maintained at the University of Birmingham. All research data will be stored on secure SQL servers at the University of Birmingham, to which only authorised users will have access. The University’s Data Protection Policy and the Conditions of Use of Computing and Network Facilities set out the security arrangements under which sensitive data should be processed and stored. All studies at the University of Birmingham are registered with the Data Protection Officer and data held in accordance with the General Data Protection Regulation. Data will be stored for a minimum of 10 years but with an expectation for storage up to 25 years.

### Confidentiality {27}

Personal data recorded on all documents will be regarded as strictly confidential and handled and stored in accordance with the General Data Protection Regulation and Data Protection Act 2018. In correspondence between the Birmingham Clinical Trials Unit and site staff, participants will only be identified using their unique trial identification number, date of birth and initials on the case report form. Participants will be asked to provide explicit consent for the transfer of a copy of their consent form (containing identifiable patient data) from the host site to Birmingham Clinical Trials Unit. This will be used to perform in-house monitoring of the consent process.

### Plans for collection, laboratory evaluation and storage of biological specimens for genetic or molecular analysis in this trial/future use {33}

Not applicable, no biological samples collected.

## Statistical methods

### Statistical methods for primary and secondary outcomes {20a}

The primary comparison groups will be those treated with PVB post operation versus those treated with TEB. All analyses will be based on the intention to treat principle, i.e. all participants will be analysed in the treatment group to which they were randomised irrespective of compliance or other protocol deviation but excluding patients that did not go on to have surgery. For all outcome measures, appropriate summary statistics will be presented by group (e.g. mean differences and relative risk) with supporting 95% confidence intervals. Intervention effects will be adjusted for the minimisation variables listed in Sect. 16a where possible. No adjustment for multiple comparisons will be made. A two-sided *p*-value of < 0.05 will be considered statistically significant. Statistical analysis will be performed using Stata version 17.

#### Primary outcome assessment

The primary outcome is the presence of CPTP at 6 months post-randomisation. A mixed effects log-binomial regression model will be used to calculate adjusted relative risks and 95% confidence intervals, adjusting for the intervention group and the minimisation variables. All minimisation variables will be treated as fixed effects, apart from the centre, which will be included as a random effect. The *p*-value from the associated chi-squared test will be produced and used to determine the statistical significance of the estimated treatment group parameter.

#### Secondary outcome assessment

Analysis on the presence of CPTP will also be performed at 3 months and 12 months, whilst all remaining secondary outcomes will be analysed at each time point, as appropriate. The presence of CPTP and mortality will be analysed similarly to the primary outcome. Questionnaire responses (VAS, BPI, EQ-5D, HADS and SF-MPQ-2) will be converted to scores and analysed using a mixed linear regression model, adjusting for the intervention group, baseline score (if available) and the minimisation variables (again, centre will be included as a random effects variable). Further supportive analyses will be carried out on questionnaire responses using a repeated measures [31] (multi-level) model incorporating all recorded scores (baseline and the three post-treatment scores). Parameters allowing for participant, intervention group, time, baseline score and the minimisation variables will be included. A random intercept component will also be included. Regarding safety, the total number of patients experiencing SAEs will be reported by intervention group along with a descriptive table of the events, and statistical significance will be determined by a chi-square test.

A separate statistical analysis plan has been produced and provides a more comprehensive description of the planned statistical analyses.

### Interim analyses {21b}

Interim analyses of safety and efficacy for presentation to, and review by, the independent DMEC will take place during the trial. The committee will meet prior to trial commencement to agree on the manner and timing of such analyses, but this is likely to include the analysis of the primary and major secondary outcomes and full assessment of safety (SAEs) at least at annual intervals. Criteria for stopping or modifying the trial based on this information will be ratified by the DMEC. These interim analyses will be prepared by the Trial Statistician and shared solely with the DMEC. The chief investigator, trial management group and site investigators will be blinded to these interim analyses.

### Methods for additional analyses (e.g. subgroup analyses) {20b}

#### Subgroup analyses

Subgroup analyses will be limited to the same variables used in the minimisation algorithm (see Sect. 16a), apart from the centre. Tests for statistical heterogeneity (e.g. by including the treatment group by subgroup interaction parameter in the statistical model) will be performed before examining effect estimates within subgroups. The results of subgroup analyses will be treated with caution and will be used for hypothesis generation only.

#### QRI analyses

Full or targeted sections of interviews and audio-recorded appointments will be transcribed verbatim by an approved transcription service and edited to ensure the anonymity of the respondent. Data will be managed using NVivo software (NVivo v12., QSR International, Daresbuty, UK). Interview data will be analysed thematically using constant comparative approaches derived from Grounded Theory methodology [32]. Audio-recorded recruitment consultations and follow-up discussions will be interpreted using thematic analysis and other QuinteT methods [33].

#### Economic analyses

In order to assess the costs and benefits of PVB compared with TEB, both a within-study and a model based economic analysis will be undertaken.

Within study analysis—This component will use the data collected within the trial; estimates of cost-effectiveness will include the primary outcome within the trial; CPTP at 6 months post-randomisation. The principal economic analysis will assess cost-effectiveness based on incremental cost per quality adjusted life year (QALY) gained at 6 months post-randomisation, with a secondary analysis of cost per case of CPTP avoided at 6 months. If sufficient data is available, this analysis will be extended to cover outcomes and resource use at 12 months post-randomisation.

Model-based analysis beyond the end-point of the trial—If the trial shows that PVB is effective in reducing CPTP compared with TEB, it will be necessary to assess the cost-effectiveness of PVB in the longer term, in order to take into account the impact of chronic pain on an individual’s quality of life and productivity. Therefore, if deemed necessary based on the trial’s results, a decision-analytic model will be used to evaluate the longer-term impacts of the different types of analgesic. As a starting point, the model development process will use other models developed for chronic pain [34, 35]. The evidence used in the model will be drawn from the trial, with data on longer term costs and outcomes derived from the literature. If data availability permits, a societal perspective will be adopted alongside a healthcare perspective.

### Methods in analysis to handle protocol non-adherence and any statistical methods to handle missing data {20c}

Every effort will be made to collect full follow-up data on all trial participants; it is thus anticipated that missing data will be minimal. Participants with missing primary outcome data will not be included in the primary analysis in the first instance. This presents a risk of bias, and sensitivity analyses will be undertaken to assess the possible impact of the risk. This will consist of simulating the missing responses using a multiple imputation approach. Parameters used to simulate the missing responses will include the minimisation variables, intervention group, the participant’s previous responses at each time point and whether the value is missing due to death or other reason. It is not anticipated that the randomised interventions will be associated with the number of deaths, i.e. missing due to death is expected to be a random event. Additional sensitivity analysis on the primary outcome will involve varying the VAS thresholds to define CPTP as VAS worst chest pain (i) greater than 30 and (ii) greater or equal to 70.

### Plans to give access to the full protocol, participant level-data and statistical code {31c}

The full protocol is publicly assessable online via the trial website [36]. The datasets generated during the current study can be made available by the chief investigator upon reasonable request and in agreement with the research collaboration and data transfer guidelines of Birmingham Clinical Trials Unit.

## Oversight and monitoring

### Composition of the coordinating centre and trial steering committee {5d}

#### Trial management group

The trial management group will be chaired by the chief investigator and include clinical trials unit, clinician, statistical, health economic, qualitative and patient and public partner representation. This group will monitor all aspects of the conduct and progress of the trial, ensure that the protocol is adhered to and take appropriate action to safeguard participants and the quality of the trial itself. This group will meet approximately monthly though it will meet more frequently as required by ongoing trial activities.

#### Trial steering committee

A single trial steering committee (TSC) will be created for the TOPIC 2 trial and meet face-to-face or via teleconference at least once prior to recruitment of the first patient, then at least annually until full publication of TOPIC 2, and as required depending on the needs of the trial office. The TSC will be led by an independent chair and, as per NIHR HTA guidelines, will be composed of an independent statistician, health economist, clinician, patient representative and observers. Membership and duties/responsibilities are outlined in the TSC charter. In summary, the TSC will provide overall oversight of the trial, including the practical aspects of the trial, as well as ensuring that the trial is run in a way which is both safe for the patients and provides appropriate data to the sponsor and investigators.

### Composition of the data monitoring committee, its role and reporting structure {21a}

A data monitoring and ethics committee (DMEC) will be led by an independent chair who is an expert in the field. As per NIHR HTA guidelines, the DMEC will be composed of an independent expert statistician and clinician. Data analyses will be supplied in confidence, and the DMEC will be asked to advise whether the accumulated data from the trial, together with the results from other relevant research, justify the continuing recruitment of further participants. The DMEC will operate in accordance with a trial specific charter based upon the template created by the Damocles Group [37]. The DMEC will meet at least annually as agreed by the committee and documented in the charter.

### Adverse event reporting and harms {22}

The safety profile for the trial population and trial interventions are well established; therefore, a strategy of targeted recording of AEs is being employed which it is believed will not affect the safety of participants. Defined, expected complications of regional anaesthesia and postoperative surgical complications (Table 4) will be collected in trial specific CRFs.

**Table 4:** Protocol defined anticipated complications of regional analgesia and surgery

Category	Complication
Complications of regional/systemic analgesia	Nausea Vomiting Post-dural puncture headache Block failure Vascular puncture Pleural puncture Hypotension Urinary retention Drowsiness Itching High Block Inadequate pain relief
Surgical complications	Bronchopleural fistula Prolonged air leak Pneumothorax Surgical emphysema Post-surgical bleed Chylothorax
Respiratory complications	Atelectasis Pneumonia ARDS Pulmonary aspiration Pulmonary embolism Pleural effusion
Cardiovascular complications	Atrial arrythmia Ventricular arrythmia Myocardial infarction Deep venous thrombosis
Renal complications	Renal failure
Neurological complications	Transient or permanent neurologic deficit Coma

Beyond these defined, expected complications, adverse events will be reported in accordance with the UK Policy Framework for Health and Social Care Research, the Principles of GCP as set out in the UK Statutory Instrument (2004/1031; and subsequent amendments) and the requirements of the Health Research Authority (HRA). When completing an SAE form, the principal investigator or medically qualified delegate will be asked to define the causality and the severity of the SAE. On receipt of an SAE form at the trial office, the chief investigator or delegate will independently determine causality of the SAE. An SAE judged by the PI, CI or delegate(s) to have a reasonable causal relationship with the intervention will be regarded as a related SAE. The CI or delegate(s) will assess all related SAEs for expectedness. If the event is unexpected, i.e. is not defined in the protocol as an expected event, it will be classified as an unexpected and related SAE.

### Frequency and plans for auditing trial conduct {23}

TOPIC 2 is a non-CTIMP which has been formally risk assessed by the sponsor as “low risk” on the basis that both interventions are already in common usage throughout the UK, and the safety profiles are well established. Therefore, on-site monitoring will be limited to the first 5 sites to recruit a patient. Additional on-site monitoring visits may however be triggered, for example by poor CRF return, poor data quality, high or low SAE reporting rates, excessive participant withdrawals or deviations, or any other aspect of trial conduct that raises concerns about quality management. Sites will be requested to send copies of signed consent forms and other documentation for central review for all participants providing explicit consent.

### Plans for communicating important protocol amendments to relevant parties (e.g. trial participants, ethical committees) {25}

All study (including protocol) amendments will be submitted for approval to the REC and HRA. Sites will be informed of all approved minor or substantial amendments and will be asked to review and confirm approval at local site level.

### Dissemination plans {31a}

The dissemination strategy will consist of three strands. The first will ensure that patients and the public are informed of the trial results; the second will engage practitioners and health care planners locally to encourage implementation of the study’s findings, and the third will consult with policymakers for maximum impact.

#### Patients and the public

The PPI representatives will help to develop a detailed dissemination plan for the trial. Public contributors will design a ‘plain English’ summary of the study findings suitable for dissemination to a non-clinical audience. PPI representatives will design presentation materials for dissemination to key stakeholder groups. In addition, we will collaborate with consumer organisations such as Cancer Research UK, the British Lung Foundation, and the British Pain Society to bring the results of this study to a large lay audience.

#### Practitioners and health care planners

Our findings will also be presented at local, national and international anaesthetic and cardiothoracic surgery meetings, which will capture a large audience of clinicians. The results of TOPIC2 will be published in an HTA monograph, which will include the clinical and cost-effectiveness aspects of the study. These will also be submitted to high-impact international journals, aimed at a general audience to ensure maximum reach.

#### Policy makers

We will approach the Royal College of Surgeons, Royal College of Anaesthetists and National Institute for Clinical Excellence. The conclusions of TOPIC2 will be directly fed back to these organisations and impact their conclusions and future recommendations for analgesia provision in patients undergoing open thoracic surgery.

## Discussion

### Impacts of COVID-19

This trial opened to recruitment on 3 January 2019. After recruiting steadily for over a year, on 24 March 2020, recruitment was paused due to the COVID-19 pandemic. Despite reopening to recruitment in July 2020, further waves of COVID-19 infection led to sporadic recruitment with many sites being unable to recruit for prolonged periods. The lingering impact of the pandemic remains with unprecedented pressure on Research and Development infrastructure, reduced capacity for elective surgery and ongoing diversion of elective services to private sector hospitals precluding trial recruitment. NHS healthcare workers strike actions added further negative impact on trial recruitment. The full effects will be discussed in the final trial publication.

## Trial status

Protocol version number: V5.0 dated 8 October 2021. This trial is recruiting having recruited its first participant on 8 January 2019. The anticipated end date for recruitment is 30 June 2023.

## Data Availability

The final data set itself will only be available to the direct TOPIC 2 Trial Team, including the TSC, in the first instance. It will however also be made available upon formal request when the reason for the request is approved by the TSC.

## Abbreviations

BPI: Brief Pain Inventory
CPTP: Chronic post-thoracotomy pain
DMEC: Data monitoring and ethics committee
GCP: Good Clinical Practice
HADS: Hospital Anxiety and Depression Scale
PPCs: Postoperative pulmonary complications
PPI: Patient and public involvement
PVB: Paravertebral blockade
QRI: QuinteT Recruitment Intervention
SF-MPQ-2: Short form McGill pain questionnaire 2
TEB: Thoracic epidural blockade
TSC: Trial steering committee
VAS: Visual analogue scale
